# Development of monoclonal antibodies against *Rhodococcus equi* virulence-associated protein N and their application to pathological diagnosis

**DOI:** 10.1128/spectrum.00729-23

**Published:** 2023-10-06

**Authors:** Yasunori Suzuki, Shinji Takai, Yuri Morizane, Kentaro Yasuda, Kei Takahashi, Toko Ishitsuka, Yukako Sasaki, Mikihiro Otsuka, Satoru Kato, Hiroo Madarame, Makoto Sugiyama, Hiroaki Kawaguchi, Tsutomu Kakuda

**Affiliations:** 1 Laboratory of Animal Hygiene, Kitasato University School of Veterinary Medicine, Aomori, Japan; 2 The Gifu Central Livestock Hygiene Service Center, Gifu, Japan; 3 Laboratory of Small Animal Clinics, Veterinary Teaching Hospital, Azabu University, Kanagawa, Japan; 4 Laboratory of Veterinary Anatomy, Kitasato University School of Veterinary Medicine, Aomori, Japan; 5 Laboratory of Veterinary Pathology, Kitasato University School of Veterinary Medicine, Aomori, Japan; Texas A&M University, College Station, Texas, USA

**Keywords:** monoclonal antibody, virulence-associated protein N, *Rhodococcus equi*, immunostaining

## Abstract

Since 2015, virulence-associated protein N (*vapN*)-positive *Rhodococcus equi* has been isolated from pyogenic lesions in ruminants in multiple countries, including Japan, supporting the widespread distribution of this pathogen in ruminants, and suggesting that VapN is responsible for the pathogenicity of this infection. However, no immunological diagnostic method has been established for this infection. In this study, we attempted to produce anti-VapN monoclonal antibodies and apply them to immunostaining methods. Mice were immunized intraperitoneally or subcutaneously with 5 µg of recombinant VapN (rVapN). After cell fusion and cloning by limiting dilution, we generated three anti-VapN antibody-producing hybridomas (4H4, 5C3, and 5G10). Next, VapN-based immunostaining was performed using a mouse model inoculated with rVapN, the JCM94-3 (VapN-producing) strain, or the JID03-27 (low-producing) strain. Positive reactions were observed in necrotic lesions in the liver and spleen, especially in the rVapN-treated and JCM94-3-treated groups. Immunostaining of goats (*n* = 2) and cattle (*n* = 3) presumed to be infected with *vapN*-positive *R. equi* was also positive in organs carrying necrotic lesions including the lungs, lymph nodes, abomasum, and ribs. The reactivity of immunostaining differed among the antibodies, with the 4H4 antibody exhibiting the best reactivity and allowing detection at low concentrations. However, the reactivity of the other two antibodies was improved by antigen retrieval treatment. Several known diseases cause necrotizing granuloma formation, and the immunological diagnostic method established in this study can be used to distinguish *R. equi* infection from similar diseases or identify infection in previously missed cases.

**IMPORTANCE:**

*Rhodococcus equi* can cause infection in ruminants, and its pathogenicity is suggested to be associated with VapN. Despite its wide distribution, no immunological diagnostic method has been developed for VapN-producing *R. equi*. Against this background, we attempted to develop monoclonal antibodies targeting VapN and assess their application in immunostaining. In the study, mice were immunized with recombinant VapN, and cell fusion and cloning by limiting dilution permitted the generation of three antibody-producing hybridomas. The utility of the antibodies produced from the hybridomas in immunostaining was demonstrated using an infected mouse model, and the antibodies were further applied to previously reported cases of *R. equi* infection in goats and cattle. Although the 4H4 antibody induced the strongest reactions, the reactivity of two other antibodies was improved by antigen retrieval. Our monoclonal antibodies will be utilized to support the definitive diagnosis of suspected *R. equi* infection, including cases that were previously missed.

## INTRODUCTION

*Rhodococcus equi* is known in the field of veterinary medicine as the causative agent of pyogranulomatous pneumonia in foals (1, 2). In addition, *R. equi* has been isolated from various animal species such as pigs, dogs, cats, cattle, goats, and humans, indicating its wide host range (3 – 5). The pathogenicity of *R. equi* is associated with the presence of a virulence plasmid (pVAP) encoding a family of virulence-associated proteins (Vaps), and the virulence plasmids are associated with host tropism (6 – 8). In ruminants, *R. equi* harboring the linear virulence plasmid pVAPN (encoding VapN and multiple homologs) was isolated from bovine lung abscess and granulomatous lymphadenitis in Europe in 2015 (9). Since 2016, the isolation of *vapN*-positive strains from abscesses of cattle and goats has been reported in Japan (10 – 12), suggesting a widespread distribution of *vapN*-positive strains in ruminants.

Ruminant *R. equi* infections caused by *vapN*-positive strains result in tuberculosis-like symptoms with multiple pulmonary masses and granulomas in the liver, kidneys, and/or lymph nodes throughout the body (10, 13). Therefore, it is necessary to distinguish this disease from bovine tuberculosis and other diseases during processes such as slaughter inspection. In addition, *vapN*-positive strains have been isolated from immunocompromised humans (such as patients with AIDS) with clinical symptoms similar to those of tuberculosis (4, 14, 15). Thus, *vapN*-positive strains should be considered important in livestock/public health as new pathogens for ruminants and humans. However, knowledge of *vapN*-positive strains is extremely limited, and diagnostic methods for emerging pathogens using immunohistochemical techniques have not been established.

Monoclonal antibodies are produced by fusing antibody-producing B lymphocytes with immortalized myeloma cells to form hybridomas, which can produce many specific antibodies in the laboratory (16). Monoclonal antibodies recognize a single epitope within an antigen, and therefore, they have higher specificity than polyclonal antibodies when used in immunological detection methods (16). In our laboratory, anti-VapA (17) and anti-VapB monoclonal antibodies (18) have been generated to evaluate the virulence of *R. equi* isolates and establish diagnostic methods. These antibodies have been used in immunological detection methods such as colony blotting, western blotting, and immunochromatography, and they have been applied in the rapid identification of *vapA*- (isolated mainly from lesions in horses) and *vapB*-positive strains (isolated mainly from lesions in pigs) (19 – 21). In addition, the anti-VapA monoclonal antibody produced in our laboratory can be used for immunostaining and histopathological immunodiagnosis (22, 23). However, no report has described the production of a monoclonal antibody against VapN. In this study, we attempted to produce anti-VapN monoclonal antibodies by improving the production process based on the issues highlighted in previous studies. Because we succeeded in selecting anti-VapN monoclonal antibody-producing hybridomas, we examined immunostaining methods for mouse infection models as well as goats and cattle that developed *Rhodococcus* infection to establish a pathological diagnosis method using this antibody.

## RESULTS

### Generation of anti-VapN antibody-producing hybridomas

**Fig 1:**
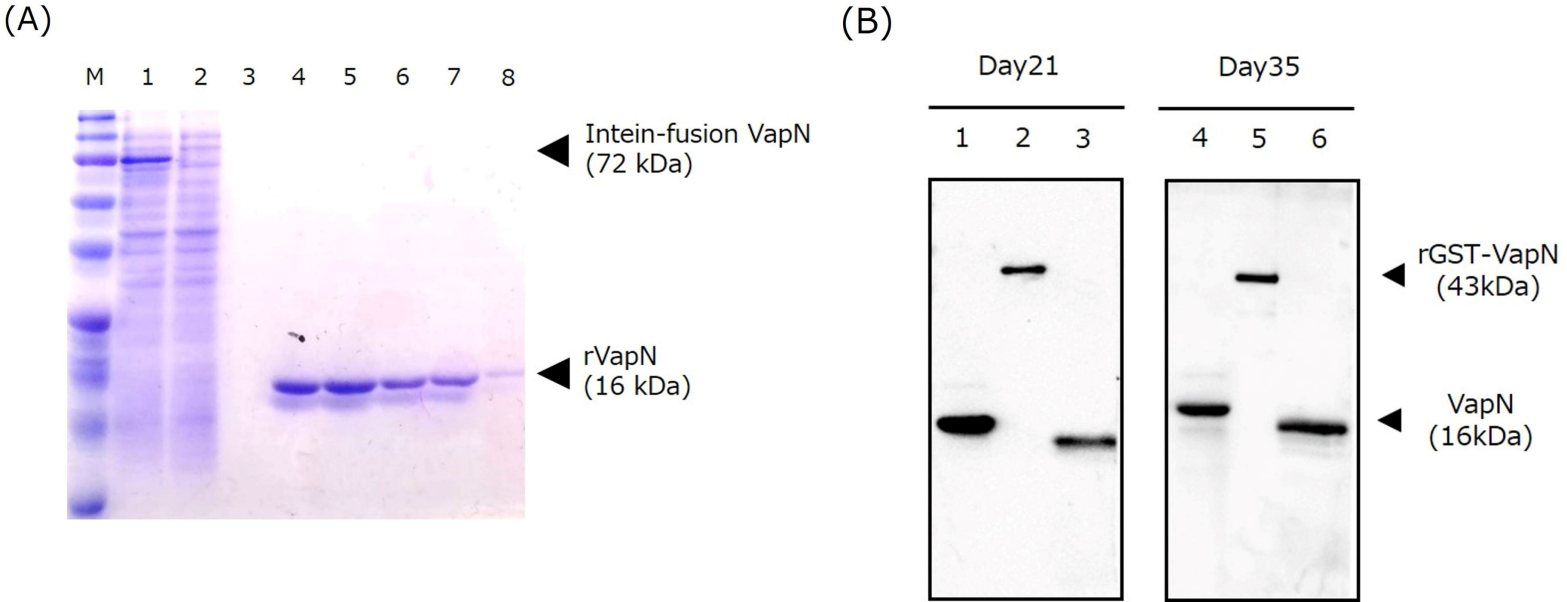
Generation of recombinant VapN protein and immunization of mice. (**A**) Purified rVapN proteins were analyzed using SDS-PAGE and detected by Coomassie blue staining. Lane 1, supernatant of sonicated *Escherichia coli* ER2566 expressing VapN by a pTYB21 vector; lane 2, flow through a chitin column; lane 3, flow through column after washing; lanes 4–8, supernatant flow through after adding elution buffer to the column; M, prestained molecular weight markers (Precision Plus Protein Dual Color Standards; Bio-Rad). (**B**) Western blot using serum from mice immunized with rVapN. After the third (day 21) and fifth (day 35) inoculations of rVapN, antibody titers against VapN were detected. Lanes 1 and 4, rVapN protein; lanes 2 and 5, recombinant GST-fusion VapN protein; lanes 3 and 6, lysate of JCM94-3 cell grown in conditioned broth (20 g/L peptone, 8 g/L NaCl, and 300 mL/L horse meat extract) adjusted to pH 6.5 for 48 h at 37°C with shaking.

Purified recombinant VapN (rVapN) subjected to sodium dodecyl sulfate-polyacrylamide gel electrophoresis (SDS-PAGE) followed by Coomassie brilliant blue staining, which detected a band around the target size of 16 kDa (Fig. 1A). BALB/c mice were immunized with this protein every other week and western blotting was performed using serum collected 21 or 35 days (immediately before cell fusion) after immunization as a primary antibody. As presented in Fig. 1B, when both rVapN and GST-fusion VapN (rGST-VapN) were used as samples, bands were detected at the expected size, namely 16 and 43 kDa, respectively. In addition, a 16 kDa band was detected when a sample of the VapN-producing JCM94-3 strain was cultured at 37°C and pH 6.5, which comprise the optimal expression condition for VapN (12, 15).

Splenocytes isolated from the immunized mice and SP2/0-Ag14 cells were fused. These fused cells (hybridomas) were seeded into 96-well plates, and enzyme-linked immunosorbent assay (ELISA) was performed using the cell culture supernatant as the primary antibody. The results demonstrated that the absorbance (A492 nm) was higher using three culture supernatants [i.e., 4H4 (A492 nm = 2.85), 5C3 (A492 nm = 3.23), and 5G10 (A492 nm = 3.06)] than that obtained using mouse serum (collected 35 days after immunization) as a positive control (A492 nm = 0.48). After cloning using the limiting dilution method, hybridomas in the wells with the highest A492 nm values for each of the three cell lines were maintained in culture (Table 1).

**TABLE 1:** Selection of cell lineages exhibiting high antibody titers for each hybridoma by limiting dilution

Hybridoma name	Wells with high values^*a*^	OD
4H4		
	**E12**	**3.40**
	E1	2.59
	B7	2.48
	D8	2.36
5C3		
	**D12**	**3.40**
	G11	3.31
	E8	3.27
	D5	2.67
5G10		
	**H3**	**3.18**
	C11	3.08
	B3	3.04
	B10	1.98
Positive control^*b*^		1.16–1.47

^*a*^ After cloning using the limiting dilution method, ELISA was performed using the culture supernatants. rGST-VapN was used as the plate-coated antigen. In total, 37 wells from 4H4 derivatives, 19 from 5C3 derivatives, and 18 from 5G10 derivatives had higher values than the positive control. The four wells with the highest OD values of a single clone that was found to grow from one hybridoma by microscopy are presented. Hybridomas in the wells with the highest OD values for the three cell lines were maintained in culture (boldface).

^*b*^ The mouse serum collected after rVapN immunization (day 35) was used as a positive control.

### Purification and specificity of anti-VapN antibodies

**Fig 2:**
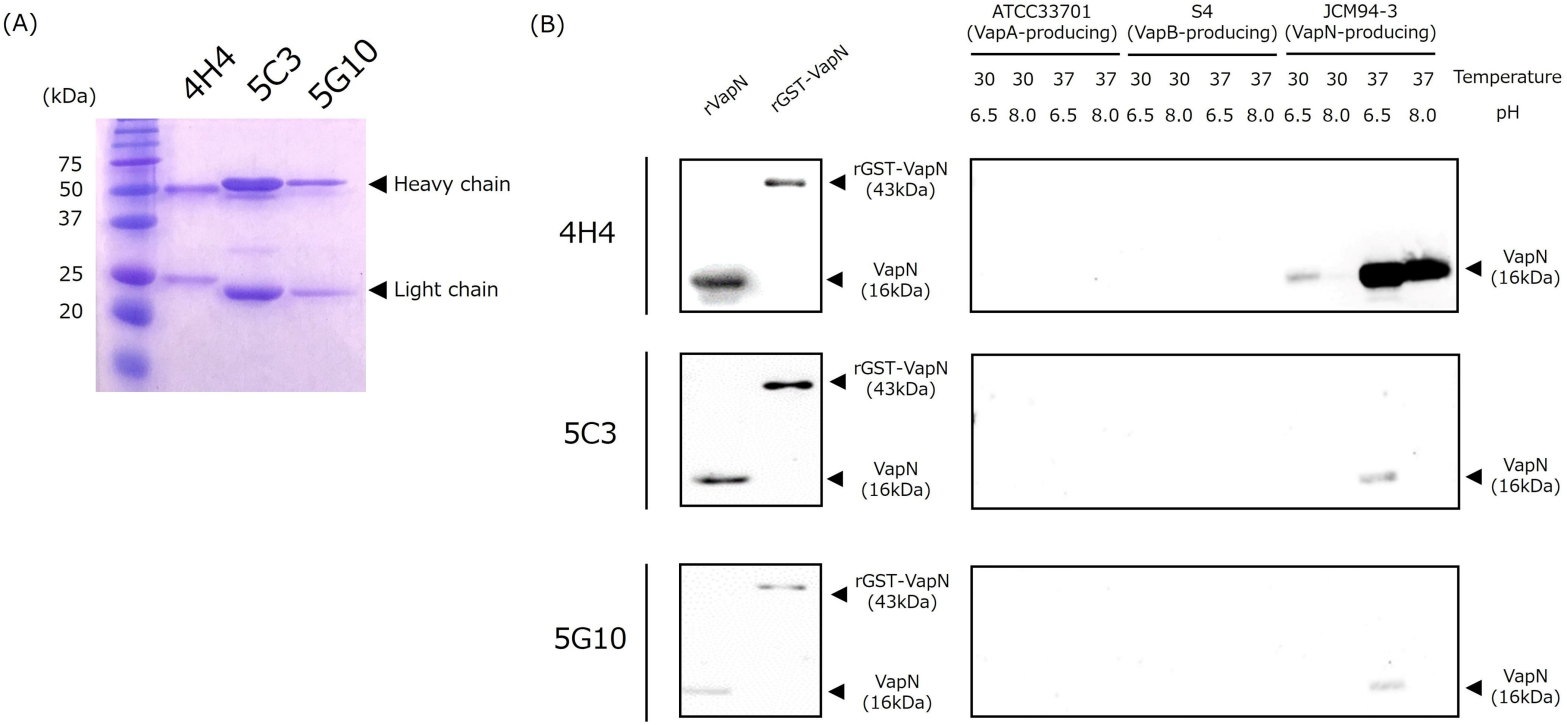
Purity and specificity of anti-VapN monoclonal antibodies. (**A**) Three purified anti-VapN monoclonal antibodies were analyzed using SDS-PAGE and detected with Coomassie staining. (**B**) Western blot using each antibody against rVapN, rGST-VapN, and cell lysates of VapA-, VapB-, and VapN-producing strains. The protein amount was adjusted to 100 ng/10 µL and subjected to electrophoresis. The preparation of the cell lysate was described in previous studies (12, 15).

After acclimation of the three hybridomas to a serum-free medium, approximately 5 mg of anti-VapN monoclonal IgG antibody was purified from 100 mL of the serum-free culture supernatant for each hybridoma. As presented in Fig. 2A, the band size of the light chain of the 4H4 antibody was larger than the other two antibodies. The subtype of all three monoclonal antibodies was IgG1 (Fig. S1). Next, to confirm the specificity of the purified anti-VapN monoclonal IgG antibodies, we subjected recombinant proteins (rVapN and rGST-VapN) and cell lysates (VapA-, VapB-, and VapN-producing strains) to western blotting. All anti-VapN monoclonal antibodies recognized rVapN, rGST-VapN, and JCM94-3-derived cell lysates, but not VapA- and VapB-producing strain lysates (Fig. 2B; S2A). Among the antibodies, 4H4 displayed the highest reactivity against the VapN antigen under the same antibody concentration and exposure time (Fig. 2B). Furthermore, we reduced the exposure time and antibody concentration compared to the conditions in Fig. 2B (4H4 antibody: 1 µg/mL, 2nd antibody: ×50,0000). As shown in Fig. S2B, the band appeared significantly stronger in the 37°C at pH 6.5 culture than in the 37°C at pH 8.0 culture. The denser bands were likely owing to antibody concentration saturation observed in Fig. 2B. Subsequently, we performed western blotting using the 4H4 monoclonal antibody on three VapN-producing strains (JCM94-3, JCM94-25, and JCM94-27), three VapN-nonproducing strains (JCM94-16, JCM94-31, and JID03-27), and one weakly VapN-producing strain (JCM94-14). As depicted in Fig. S2C, bands were detected in JCM94-3, JCM94-25, and JCM94-27. However, JCM94-14 exhibited a relatively thinner band, and there were no bands in the remaining three strains. These results are consistent with the previous VapN-immunoserum findings (15).

### Immunostaining in a mouse model

**Fig 3:**
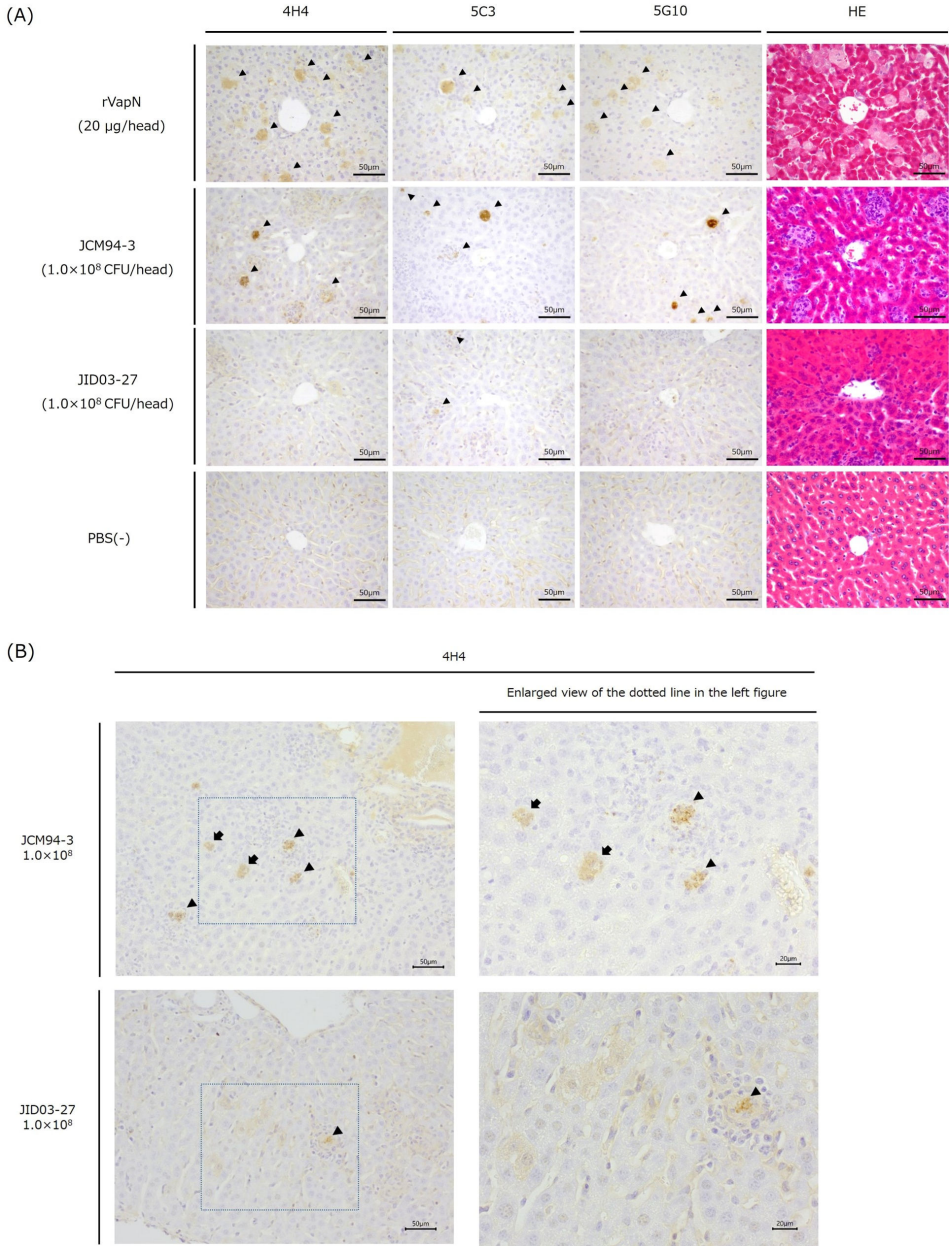
Investigation of VapN-immunostaining methods using a mouse model. (**A**) Pathological changes in the liver after the inoculation of rVapN, a VapN-producing strain (JCM94-3), or a low VapN-producing strain (JID03-27). Positive reactions are indicated by an arrowhead. (**B**) Magnified image of VapN immunostaining in the liver after the inoculation of JCM94-3 or JID03-27. The 4H4 monoclonal antibody was used as the primary antibody. Positive reaction detected within enlarged necrotic hepatocyte is indicated by an arrow, and within microgranuloma formation is indicated by an arrowhead, respectively. The scale bar is as presented in the figure.

Considering the pathological findings of the hematoxylin and eosin (HE)-stained images of rVapN-administered mice, hepatocytes were diffusely enlarged and necrotic. Comparing the central venous and portal venous regions, more enlarged hepatocytes were observed in the portal venous region (Fig. 3A). The spleen featured many “starry sky-like patterns” in the white pulp (Fig. S3). In HE-stained image of mice inoculated with JCM94-3, enlarged necrotic hepatocytes like rVapN-administered mice and microgranuloma formations were observed in the liver (Fig. 3A). In the spleen, “starry sky-like patterns” were observed similar to the findings with rVapN-administered mice (Fig. S3). In HE-stained images of mice inoculated with JID03-27 (low VapN-producing strain), focal microgranulomas were detected in the liver, but they were not as pronounced in the mice inoculated with rVapN or JCM94-3 and the tissue structure was like that of normal tissue (Fig. 3A). In the spleen, HE-stained images showed that almost no starry sky-like patterns were observed in the white pulp, and the tissue structure was similar to that of normal tissue (Fig. S3). No pathological changes were observed in the PBS (−)-administered mice, and a normal tissue structure was maintained (Fig. 3A; Fig. S3).

To apply the anti-VapN monoclonal antibody to the pathological examination of animals infected with VapN-producing strains, immunostaining was examined using a mouse infection model. When rVapN was administered, diffusely positive immunostaining was observed mainly in the enlarged hepatocytes (Fig. 3A) and the center of “starry sky-like patterns” in the spleen (Fig. S3). Similarly, when the VapN-producing strain JCM94-3 was administered, diffusely positive VapN-immunostaining reactions were detected in the enlarged hepatocytes (arrow in the upper images in Fig. 3B), in the center of microgranuloma (arrowhead in the upper images in the Fig. 3B), and in the center of “starry sky-like patterns” in spleen (Fig. S3). When JID03-27 (low VapN-producing strain) was administered, immunostaining for VapN shows that slight positive reactions were detected in the microgranulomas (arrowhead in the lower images in Fig. 3B). In the PBS (−)-administered mice, no positive reaction was observed in almost all sections of either organ. However, positive reactions were scattered in the border region between the white pulp and red pulp of the spleen, suggesting that the positive reactions were nonspecific (Fig. S3). We also immunostained using commercially available mouse IgG for the primary antibody. In the liver, we confirmed that there was no positive reaction in enlarged hepatocytes and microgranuloma. In the spleen, we confirmed that there was no positive reaction in the center of “starry sky-like patterns” stained when monoclonal antibodies were used (Fig. S4).

### Immunostaining of *R*. *equi* infection in ruminants

**Fig 4:**
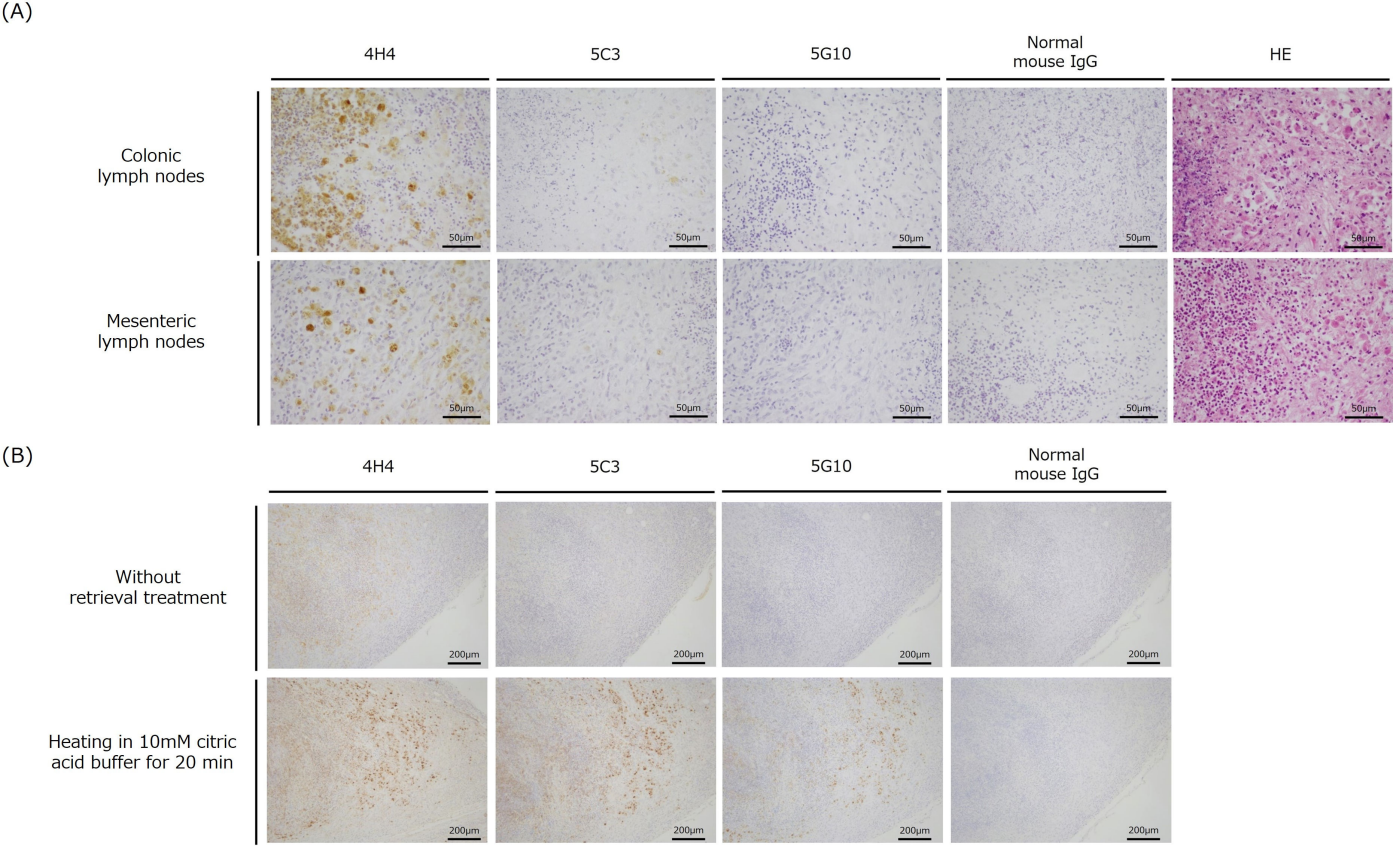
Investigation of VapN immunostaining in the pyogenic inflamed colon and mesenteric lymph nodes in a case of *R. equi* infection in a goat in Okinawa, Japan in 2015. (**A**) Immunostaining images of three different VapN monoclonal antibodies used as primary antibodies. Each antibody was adjusted to 2 µg/mL. (**B**) Alteration in the stainability of lymph node lesions attributable to antigen retrieval treatment. Before immunostaining, the sections were heated in 10 mM citrate buffer for 25 min. Each antibody was adjusted to 2 µg/mL. The scale bar is as presented in the figure.

First, colonic and mesenteric lymph nodes collected from goat cases that occurred in 2015 in Okinawa, Japan (11) were subjected to immunostaining. When the 4H4 antibody was used, strong positive reactions were observed in sections of these lymph nodes (Fig. 4A). When the 5C3 antibody was used, sections of all organs exhibited a slightly positive reaction in a localized manner compared to the findings reported with the use of 4H4 antibody (Fig. 4A). On the contrary, when the 5G10 antibody was used, no positive reaction was observed in any organ (Fig. 4A). When these sections were subjected to epitope retrieval of the antigen protein by heating, a strong positive reaction was detected in the same region with all three antibodies (Fig. 4B).

**Fig 5:**
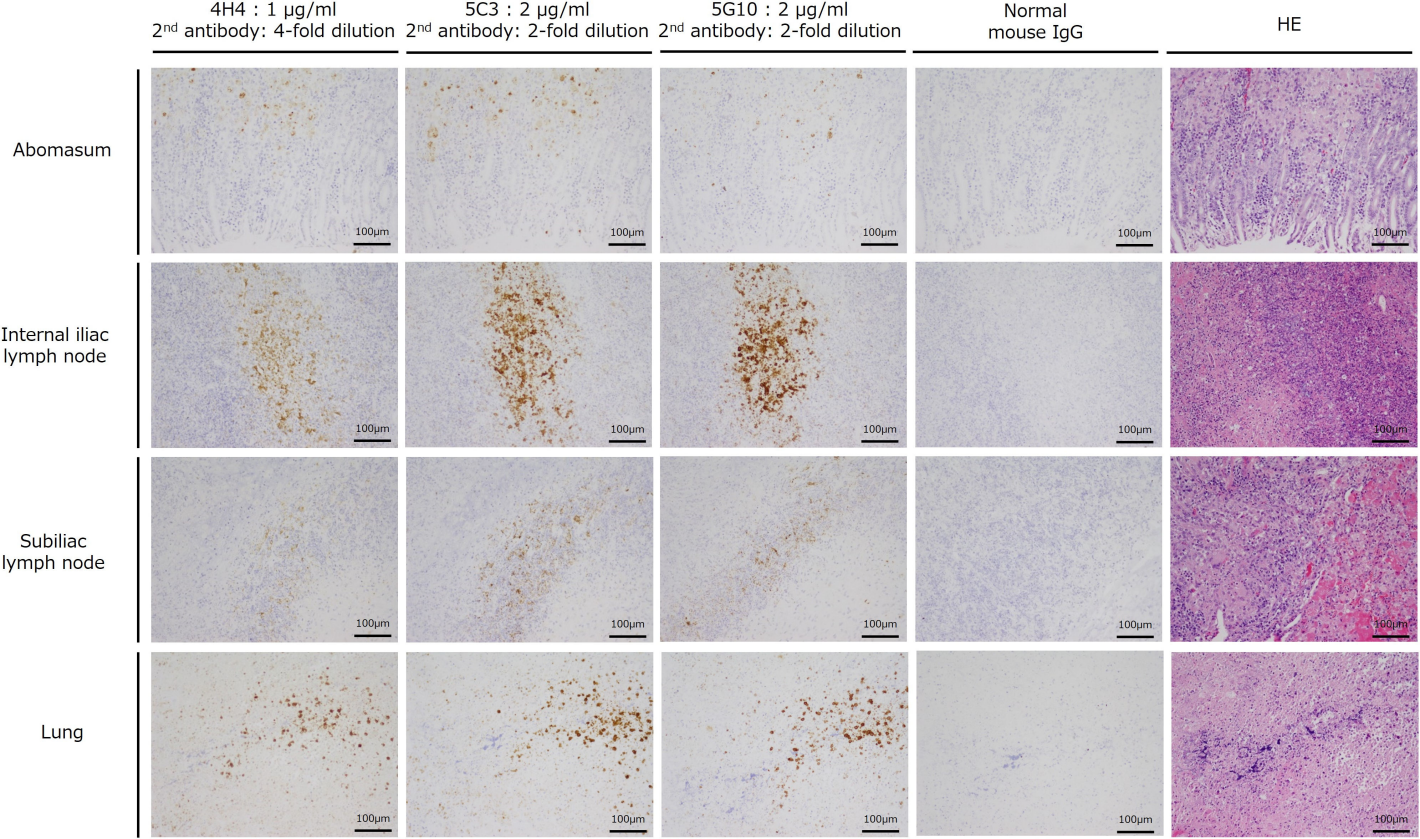
Investigation of VapN immunostaining in the abomasum, lymph nodes, and lungs in a case of *R. equi* infection in cattle in Oita, Japan. All sections were immunostained after heat treatment for epitope retrieval. All sections were immunostained following epitope retrieval, which was achieved by heating thin sections in 10 mM citrate buffer (pH 6.0) for 25 min at 95°C using KOS Microwave HistoSTATION (Millestone Srl, Sorisole, Italy). When staining with the 4H4 monoclonal antibody, the concentrations of both the primary and secondary antibodies were halved (i.e., 1 µg/mL for the former and 4× commercial dilution for the latter). The scale bar is as presented in the figure.

Next, eight organs (heart, kidneys, liver, abomasum, spleen, internal iliac lymph nodes, subiliac lymph nodes, and lungs) from infected cattle identified in Oita, Japan were subjected to immunostaining. Because no positive reaction was detected in antigen-untreated sections of this individual using any of the antibodies, immunostaining of sections subjected to epitope retrieval of the antigen protein by heating was examined. As shown in Fig. 5, positive immunostaining reactions were observed in the abomasum, internal iliac lymph nodes, subiliac lymph nodes, and lungs for all three antibodies. The concentrations of primary and secondary antibodies were also optimized in the immunostaining of this section. The results revealed that the antibody reactivity was best for 4H4, followed by 5C3 and 5G10 in that order. Namely, 4H4 (primary antibody, 1 µg/mL; secondary antibody, 4× dilution) exhibited better reactivity at a lower concentration than 5C3 (primary antibody, 2 µg/mL; secondary antibody, 2× dilution) or 5G10 (primary antibody, 2 µg/mL; secondary antibody, 2× dilution).

### Application to past and recent cases in ruminants

**Fig 6:**
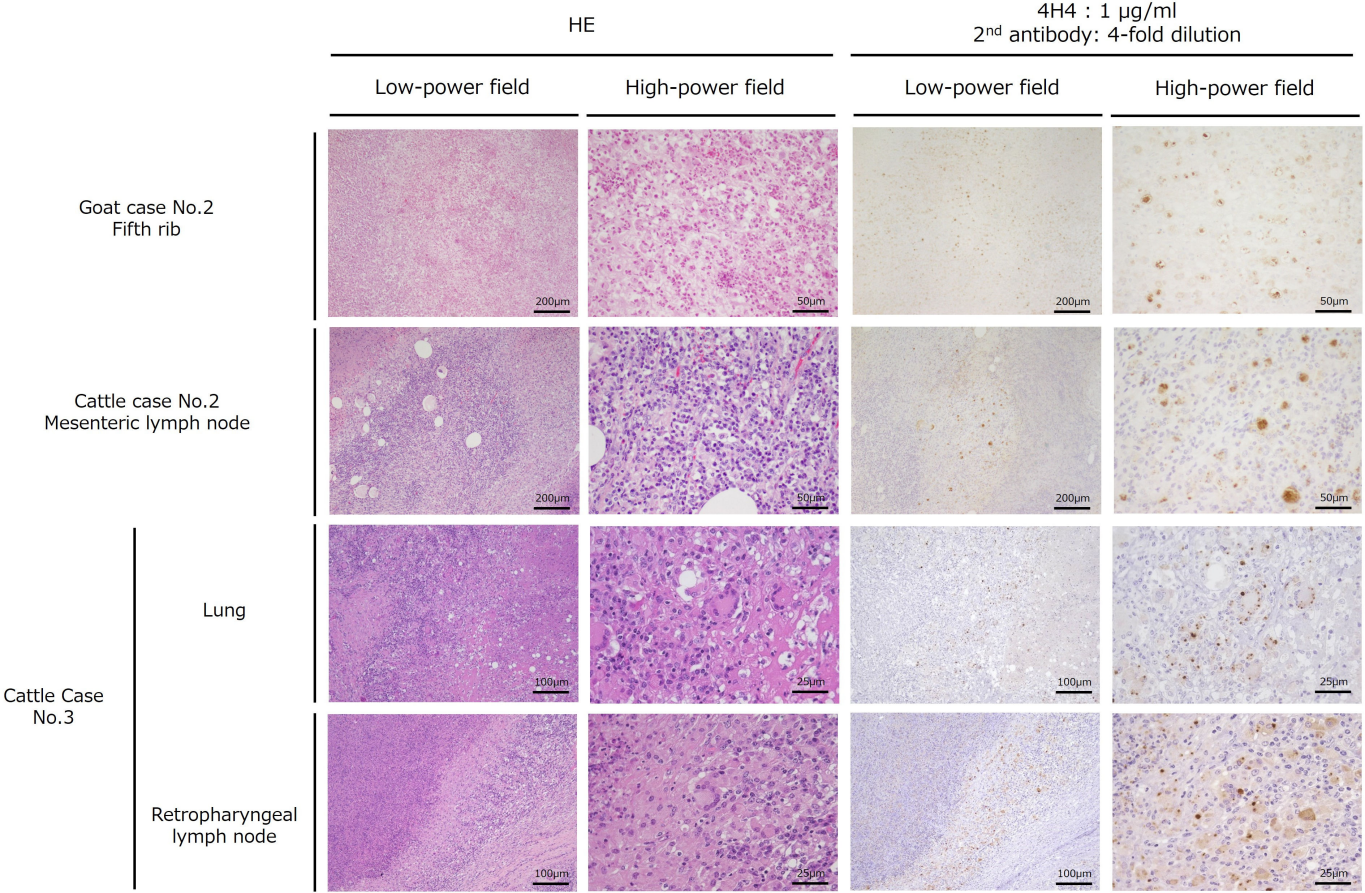
Application of VapN immunostaining to cases of *R. equi* infection in ruminants. The fifth rib was used as the specimen in goat case No. 2, the mesenteric lymph node was used in cattle case No. 2, and the retropharyngeal lymph nodes and lungs were used in cattle case No. 3. Details of these cases are presented in Table S1. All sections were immunostained after heat treatment for epitope retrieval. All sections underwent immunostaining after epitope retrieval. This was achieved by heating the thin sections in 10 mM citrate buffer (pH 6.0) for 25 min at 95°C using KOS Microwave HistoSTATION (Millestone Srl, Sorisole, Italy). All primary antibodies used for immunostaining were 4H4 monoclonal antibodies (1 µg/mL). The scale bar is as presented in the figure.

We attempted to adapt the aforementioned immunostaining method (4H4 as a primary antibody, 1 µg/mL; secondary antibody, 4× dilution) to cases in goats occurring at Michigan State University in 1998 (24) and cattle cases occurring in Aichi and Gifu prefectures in Japan in 2022. In goat case No. 2, the intramedullary cavity contained an abscess surrounded by radially arranged perpendicular spicules of new periosteal bone in the fifth right rib, and immunostaining with the anti-VapA monoclonal antibody 10G5 was negative (24). A section of the aforementioned right fifth rib was stained, and coccobacilli in macrophages in the bone marrow were stained with hematoxylin and positive in VapN immunostaining (top images in Fig. 6). Cattle case No. 2 occurring in Aichi in 2022 involved a 14-month-old black Japanese breed with systemic signs of inflammation and swelling of lymph nodes and abscess formation throughout the body. Cattle case No. 3 occurring in Gifu in 2022 involved a 43-month-old Japanese black breed with multiple abscesses in the subcutaneous neck, lungs, posterior pharyngeal lymph nodes, mediastinal lymph nodes, and mesenteric lymph nodes. In the lymph nodes of both cases and sections of the lungs of case No. 3, positive reactions were detected within scattered epithelioid cells and many clustered multinucleated giant cells surrounding the central necrotic foci, as observed in the aforementioned case in Oita (images of the second to fourth rows in Fig. 6).

## DISCUSSION

To evaluate the pathogenicity of *R. equi* isolates and establish diagnostic methods, we produced anti-VapA (17) and anti-VapB monoclonal antibodies (18). These monoclonal antibodies have been used in immunological detection methods for the rapid identification of VapA- and VapB-producing strains. Conversely, since the discovery of VapN in 2015 (9), the isolation of VapN-positive *R. equi* from abscess lesions in goats and cattle has been reported domestically and internationally, suggesting a widespread distribution in ruminants. However, there is no report of the production of monoclonal antibodies against VapN that can be applied to the diagnosis of infection at this time. Therefore, this study aimed to produce an anti-VapN monoclonal antibody and apply it to the diagnosis of infection by VapN-producing strains.

By fusing SP2/0-Ag14 cells and splenocytes from mice with elevated antibody titers, we finally succeeded in generating three hybridoma lines (4H4, 5C3, and 5G10). We believe that we have established a simpler, shorter, and more stable purification method than the conventional method of inoculating hybridomas into nude mice (25) and purifying them from ascites fluid because we can obtain anti-VapN monoclonal antibodies simply by culturing hybridomas for several days. The three antibodies exhibited differences in reactivity against the rVapN protein and VapN-producing strain under the same conditions, with the 4H4 antibody exhibiting the best reactivity. In addition, the SDS-PAGE of each anti-VapN monoclonal antibody revealed that 4H4 had a different light chain size than the other two antibodies. These results suggest that 4H4 is structurally different from the other two antibodies in terms of the epitopes it recognizes. To identify the epitope of each antibody, the amino acid sequence of the mature VapN protein (152 amino acids) was divided into six parts, and each artificial synthetic peptide (amino acids 1–30, 25–54, 49–78, 73–102, 98–127, and 123–152) was used as a solid-phase antigen for ELISA epitope mapping similarly as previously described for VapA (26). However, no clear reaction was observed using each antibody (Fig. S5). The exact reason for this outcome remains unknown, but it is possible that epitopes recognized by each monoclonal antibody are located within mechanically determined peptide fragments (e.g., straddling amino acids 24–32). Another possibility is that the peptide antigens used in this study were not solidified as effectively as expected.

If antibodies with different epitopes could be produced, then they could be applied to immunological detection methods that use two different antibodies, such as sandwich ELISA and immunochromatography. Further studies are needed to determine the epitopes of these three antibodies.

To apply the anti-VapN monoclonal antibody to the pathological examination of VapN infection, we first validated it using existing mouse models. When rVapN was administered, enlarged necrotic hepatocytes were observed diffusely, and these necrotic cells exhibited a strong positive reaction in VapN immunostaining. In addition, more immunostaining-positive hepatocytes were observed in the portal venous region. These results indicated that the rVapN protein causes single-cell necrosis that accumulates hematogenously. It is considered that the toxic mechanism of rVapN in cells is caused by swelling attributable to water expansion, unlike the degeneration for ground-glass opacity (increases smooth endoplasmic reticula and microbodies in the liver cytoplasm) observed in pesticide-induced hepatotoxicity (27, 28). The results also demonstrated that rVapN treatment caused necrosis of large cells in the spleen. These were larger in diameter than lymphocytes, suggesting that rVapN was incorporated into reticulocytes and that the recombinant protein was the cause of necrosis. In our preliminary experiments, when 20 µg of rVapN was administered to mice weighing approximately 25 g (0.8 mg/kg), almost all mice died within 24 h. Therefore, the immunizing dose in this experiment was reduced, and the route of administration was divided into intraperitoneal and subcutaneous. The dose of 0.8 mg/kg was lower than the lethal dose of other bacterial toxins in mice (29). These results indicate that the VapN protein can induce cell necrosis and subsequently lead to mouse lethality. In the future, we should observe intracytoplasmic organelles to more precisely elucidate the mechanism of cellular degeneration and necrosis caused by rVapN.

In mice inoculated with VapN-producing strains (JCM94-3), microgranulomas and single-cell necrosis of hepatocytes were observed in the liver, and the pronounced positive VapN immunostaining was observed in the center of both lesions. It was considered that single-cell necrosis was induced in hepatocytes that accumulated the high VapN-producing strain, and over time, inflammatory cells accumulated around the single-cell necrotic hepatocytes, resulting in an immune response in which microgranulomas were formed. A starry sky-like image was observed in the spleen with positive immunostaining in the center of the image. Because each organ was sampled on the third day after inoculation with the JCM94-3 strain, it is not possible to fully observe the pathogenesis caused by this strain. In the future, it will be necessary to collect samples over time after administration of the pathogen to investigate the pathogenesis of each organ caused by the high VapN-producing strain. Conversely, in mice inoculated with the low VapN-producing strain (JID03-27), fewer microgranulomas and less single-cell necrosis were observed compared to the findings in mice inoculated with rVapN or JCM94-3, and a normal structure was maintained. These pathological findings of JCM94-3 and JID03-27 were consistent with our previous report (15), and in this study, it was possible to identify the cells in which VapN and its producing strains were localized by immunostaining.

Because the efficacy of immunostaining with anti-VapN monoclonal antibodies was suggested in a mouse model, we investigated whether these antibodies could be adapted to ruminant specimens with *R. equi* infection. In a case of goat infection occurring in Okinawa, Japan, a strongly positive reaction was observed in colonic and mesenteric lymph nodes when the 4H4 antibody was used. However, the reaction was weaker when the 5C3 or 5G10 antibody was used. These differences in reactivity were improved by antigen retrieval in the sections. In addition, positive reactions were not detected in cattle sections without antigen retrieval treatment using any of the antibodies. Thus, the 4H4 antibody exhibited better reactivity at lower concentrations than the other two antibodies, in line with the obtained results of western blotting. These results suggest that when immunostaining is performed on actual cases, antigen retrieval should be performed, and the 4H4 antibody should be used first. In general, during the process of tissue fixation and paraffin embedding, crosslinking reactions can occur, which may mask protein epitopes and prevent the antibody binding to the antigen. To reexpose such epitopes, antigen retrieval is used. The variation in the necessity for antigen retrieval among specimens may be due to slight differences in the time from tissue collection to fixation during autopsy, as well as potential variations in the details of the tissue fixation and paraffin embedding processes performed by different coauthors, despite following the same overall procedure described in the Materials and Methods section. It has been previously observed that the type of fixative and duration of fixation can influence antigen reactivity (30). We strongly believe that antigen retrieval is crucial in all cases and recommend its implementation when conducting immunostaining with our monoclonal antibodies.

Common pathological findings in the goats included pyogranulomatous lesions in the liver and lungs (24, 31). In addition to these symptoms, case No. 2 of goats displayed osteomyelitis in limbs, including the right humerus (24), and VapN immunostaining of the fifth rib revealed strong reactivity in the macrophages in the bone marrow. There have been several reports of osteomyelitis in goats involving the isolation of *vapN*-positive *R. equi* from vertebrae and humeral abscess formation sites (32). In *vapN*-positive *R. equi* infection, in addition to localized necrotic lesions, orthopedic signs such as lameness associated with bone lesions should be noted.

Unlike bovine tuberculosis caused by *Mycobacterium bovis*, which can never be observed by HE staining, ruminant *R. equi* can be identified as coccobacilli in macrophages by HE staining and may be differentiated from bovine tuberculosis on HE-stained sections. However, it requires skill to read and cannot be differentiated from the organism in pyogenic granulomatous lesions caused by other intramacrophage pathogens. Some diseases are known to involve the formation of pyogranulomatous lesions in the lungs, liver, and lymph nodes. For example, *Corynebacterium pseudotuberculosis* infection is one such disease that leads to disseminated granulomas in ruminants. This infection is highly contagious, and it can result in serious economic losses caused by reduced production efficiency and carcass disposal, making it an extremely serious infection in ruminants (33). The immunological diagnostic method established in this study is expected to be used to differentiate *R. equi* infection from similar diseases. In particular, it would be valuable for veterinary pathologists who regularly conduct differential diagnoses of livestock diseases (e.g., the pathology section at the Livestock Hygiene Service Center, a government agency in Japan). In addition, there have been a series of cases of *vapN*-harboring *R. equi* infection in ruminants in Japan since 2016 (5, 10). We hope that our monoclonal antibodies will be utilized to support the definitive diagnosis of suspected *R. equi* infection, including cases that were previously missed.

## MATERIALS AND METHODS

### Bacterial strains

The *R. equi* strains used in this study are presented in Table 2. Both *Escherichia coli* NEB 10-beta Competent Cells and ER2566 Competent Cells were purchased from New England Biolabs (Ipswich, MA, USA).

**TABLE 2:** *Rhodococcus equi* strains used in this study

Strain name	Source	*vap* gene	Description	Reference
ATCC33701	A pneumonic foal	*vapA*	VapA-producing strain	(34)
U19	Tracheal lavage fluid of a Friesian horse	*vapA*	VapA-producing strain	(35)
S4	Mandibular lymph node of a pig	*vapB*	VapB-producing strain	(36, 37)
JCM94-3	AIDS patients in Switzerland	*vapN*	VapN-producing strain	(14, 15)
JID03-27	AIDS patients in Thailand	*vapN*	Strain carrying the VapN gene but not producing VapN protein	(14, 15)
JCM94-25	Renal transplant patients in the USA	*vapN*	VapN-producing strain	(14, 15)
JCM94-27	Lymphosarcoma patients in New Zealand	*vapN*	VapN-producing strain	(14, 15)
JCM94-14	AIDS patients in Australia	*vapN*	Weakly VapN-producing strain	(14, 15)
JCM94-16	AIDS patients in the USA	*vapN*	Strain carrying the VapN gene but not producing VapN protein	(14, 15)
JCM94-31	AIDS patients in the USA	*vapN*	Strain carrying the VapN gene but not producing VapN protein	(14, 15)

### Expression of rVapN

*R. equi* JCM94-3 was grown in BHI broth (Becton Dickinson, Franklin Lakes, NJ, USA) for 16 h at 37°C with shaking (120 rpm). Genomic DNA (gDNA) was extracted from the cultured cells using a QIAamp DNA Mini kit (Qiagen GmbH, Hilden, Germany). The concentration and purity of the extracted DNA were determined using the Qubit dsDNA HS Assay Kits (Thermo Fisher Scientific, Waltham, MA, USA) and a NanoDrop OneC spectrophotometer (Thermo Fisher Scientific), respectively. To construct the rVapN expression plasmid, forward (GTTGTTGTACAGAACCAGCCGCTGGACGTT) and reverse PCR primers (ACCTGCAGGGAATTCCTACGCCCAGCTGCC) were designed to amplify a fragment of *vapN* corresponding to the mature protein. In VapA, the homolog of VapN, there is a known Ala-rich signal sequence at the N-terminus (38). The cleavage site of the N-terminal signal peptide sequence of VapN was predicted between positions 31 and 32 (Ala-Gln) using the SignalP 4.1 Server (http://www.cbs.dtu.dk/services/SignalP/). The *vapN* fragment was amplified from gDNA by PCR using Tks Gflex DNA Polymerase (Takara Bio, Shiga, Japan). Another DNA fragment was prepared by *sap*I and *EcoR*I (New England Biolabs) digestion of pTYB21 (New England Biolabs) and purification after agarose gel electrophoresis using a QIAquick Gel Extraction Kit (Qiagen GmbH). pTYB21 is an *E. coli* expression system of the target protein to be fused to the intein tag. This fusion protein can release the target protein from the chitin resin-bound intein tag due to the self-cleavage activity of intein under reducing conditions. The two DNA fragments were then ligated with NEBuilder HiFi DNA Assembly Master Mix (New England Biolabs) and directly transformed into 10-beta Competent Cells to generate pTYB21::vapN. Nucleotide sequences were verified using an ABI 310 capillary sequencer (Thermo Fisher Scientific) and BigDye Terminator v1.1 Cycle Sequencing Kit (Thermo Fisher Scientific). *E. coli* ER2566 was transformed with pTYB21::vapN. rVapN expression and purification were performed using an IMPACT kit (New England Biolabs) according to the manufacturer’s instructions. The presence of the purified rVapN protein was determined by SDS-PAGE, and protein concentrations were measured using a Quick Start Bradford Protein Assay Kit (Bio-Rad Laboratories, Hercules, CA, USA). The GST-fusion VapN protein (rGST-VapN) was generated as previously reported (12).

### Immunization of mice and western blotting using mouse serum

BALB/cCrSlc mice were purchased from Japan SLC, Inc. (Shizuoka, Japan). Mice were housed at 22°C–25°C under a 12 h:12 h light:dark cycle. Animal experiments were approved by the Animal Research Ethics Committees of Kitasato University School of Veterinary Medicine (permit number: 21–029) and conducted in accordance with the guidelines for animal experimentation. Every other week, mice were immunized via the intraperitoneal or subcutaneous inoculation of 5 µg of rVapN protein mixed with Freund’s incomplete adjuvant (FUJIFILM Wako Pure Chemical, Osaka, Japan). After the third (day 21) and fifth (day 35) antigen inoculations, blood was collected from mice, and anti-VapN antibody titers were detected by western blotting (15). Briefly, 100 ng of rVapN or rGST-VapN was separated *via* SDS-PAGE and then transferred to polyvinylidene difluoride membranes (GE Healthcare) using a HorizeBLOT 4M-R (ATTO Corporation, Tokyo, Japan). Each test mouse serum sample (×1,000) was used as a primary antibody, and peroxidase-conjugated goat IgG to mouse IgG (Whole Molecule) (×10,000; MP Biomedicals, Santa Ana, CA, USA) was used as the secondary antibody. Signals were detected using an iBright FL1000 Imaging System (Thermo Fisher Scientific) and ECL Prime Western Blotting Detection Reagent (GE Healthcare).

### Cell cultures and cell fusion

The mouse myeloma cell line SP2/0-Ag14 (RCB0209) was provided by the Riken Bioresources Center (Tsukuba, Japan) through the National Bio-Resource Project of MEXT Japan. The cells were cultured in RPMI 1640 (Nacalai Tesque, Kyoto, Japan) containing 10% fetal bovine serum (FBS; Corning, Corning, NY, USA) in a humidified 5% CO_2_ atmosphere at 37°C. The procedures for cell fusion and purification of hybridomas were described previously (17, 18). Briefly, 35 days after the start of immunization, the mice were sacriﬁced, the shredded spleen was placed in a nylon mesh filter, and splenocytes were released by applying gentle pressure. Next, red blood cells were lysed in RBC buffer (0.15 M NH_4_Cl, 1 mM KHCO_3_, 0.1 mM ethylenediaminetetraacetic acid). The cells were counted and fused with SP2/0-Ag14 cells at a 1:1 ratio using freshly prepared 50% polyethylene glycol solution (Nacalai Tesque). After extensively washing the fusion mixture with RPMI 1640 containing 15% FBS, the cells were suspended in HAT medium [RPMI 1640 containing HAT supplement (MP Biomedicals) and 15% FBS] and seeded in 96-well microplates at 2.5 × 10⁵ cells/well. The HAT medium was changed every 2 days, and cells were grown until 80% confluent.

### Enzyme-linked immunosorbent assay

To select anti-VapN antibody-producing hybridomas, ELISA was performed using a previously published method with some modifications (12). ELISA was performed in 96-well Sumilon multi-well plates (Sumitomo Bakelite Co., Ltd., Tokyo, Japan). rVapN or rGST-VapN was diluted in phosphate-buffered saline (PBS) (−) and immobilized at 100 ng/well at 4°C overnight. After blocking with Block Ace (KAC Co., Kyoto, Japan), 100 µL of the test culture supernatant of hybridomas was added to each well. After incubation for 1 h at 37°C, 100 µL of the peroxidase-conjugated goat IgG to mouse IgG (Whole Molecule; dilution, ×4,000) was added to each well. After incubation for 1 h at 37°C, 100 µL of OPD substrate reagent (FUJIFILM Wako Pure Chemical) was added and incubated for 15 min at 37°C, and the reaction was stopped by the addition of 3 NH_2_SO_4_. A492 nm was measured using the Infinite 200 PRO (Tecan, Mannedorf, Switzerland).

### Cloning of hybridomas and acclimation to serum-free medium

The limiting dilution method was used for hybridoma cloning. Briefly, hybridomas from 96-well microplates with high antibody titers in the culture supernatant were collected by pipetting and suspended in HT medium [RPMI 1640 containing HT supplement (MP Biomedicals) and 15% FBS]. The suspension was adjusted to 1 cell/well and inoculated into 96-well plates, and the HT medium was replaced every 2–3 days. The culture supernatant was collected from the wells in which cells grew until reaching 80% confluency, and the antibody titer was determined again by ELISA. This procedure was repeated three times for cloning. Finally, from the wells with the highest titers (4H4, 5C3, and 5G10 cells), the culture was scaled up to 25 cm^2^ flasks. Hybridomas that could be continuously maintained in 25 cm^2^ flasks were acclimated to Hybridoma Serum-Free Medium (FUJIFILM Wako Pure Chemical). Serum-free medium was used as the basic medium, the FBS concentration was gradually reduced to 5%, 1%, and 0.5% at the time of passaging to allow maintenance culture, and the hybridomas were finally grown in serum-free medium alone.

### Purification of anti-VapN monoclonal antibodies from culture supernatants

Antibody purification from hybridoma culture supernatants was performed using MonoSpin ProG (GL Sciences Inc., Tokyo, Japan) according to the manufacturer’s instructions. Specifically, 5 mL of binding buffer [50 mM sodium phosphate buffer, 150 mM NaCl (pH 7.0)] was added to the MonoSpin ProG spin column, and the column was centrifuged at 1,500 × *g* for 2 min. The serum-free culture supernatant was filtered through a 0.2-µm filter, added to the column, and centrifuged at 1,500 × *g* for 2 min. Next, 5 mL of washing buffer [50 mM sodium phosphate buffer, 1 M NaCl (pH 7.0)] was added, and the mixture was centrifuged at 1,500 × *g* for 2 min. The spin column was attached to a new 50 mL tube containing neutralizing buffer [1 M Tris HCl buffer (pH 8.5)], 5 mL of elution buffer [100 mM glycine HCl buffer (pH 2.5)] was added, and the tube was centrifuged at 1,500 × *g* for 2 min. The concentration and purity of the anti-VapN monoclonal antibodies were determined by the Bradford method and SDS-PAGE. The IgG subclass of each purified antibody was determined using a Mouse Monoclonal Antibody Isotyping Test Kit (Bio-Rad). The specificity of the anti-VapN monoclonal antibody was confirmed by western blotting. The purified anti-VapN monoclonal antibody was used as a primary antibody at 2 µg/mL.

### Immunostaining using a mouse model

JCM94-3 or JID03-27 was cultured for 48 h at 30°C, adjusted to 1.0 × 10^8^ CFU per 200 µL, which is the inoculation volume, and administered to each mouse *via* the tail vein (39). In addition, 20 µg of rVapN was also administered to each mouse *via* the tail vein. Infected mice were housed for 3 days. Spleens and livers were collected from mice and fixed in 4% paraformaldehyde (pH 7.4; Sigma-Aldrich Co., St. Louis, MO, USA) for 17 h. Tissues were processed by routine methods and embedded in paraffin wax. Sections were stained with HE and immunostained with anti-VapN antibody. The primary antibody was anti-VapN monoclonal antibody (2 µg/mL), and labeled antigen was detected using a Histofine Simple Stain MAX PO (anti-mouse IgG) kit (Dilute kit 1: 4; Nichirei Biosciences, Tokyo, Japan). The antibody was visualized using 3,3′-diaminobenzidine (Nichirei Biosciences), and slides were counterstained with Mayer’s hematoxylin as described previously (40).

### Application of immunostaining to cases of the *R. equi* infection in ruminants

We verified the applicability of immunostaining in five cases in which *vapN*-harboring *R. equi* was isolated from various organs of goats (*n* = 2) and cattle (*n* = 3) displaying clinical symptoms such as loss of energy, pneumonia, and drainage of pus. A summary of each case and organs used for immunostaining is presented in Table S1. HE staining and VapN immunostaining were performed according to the aforementioned method with some consideration of the antibody concentration and pretreatment for antigen retrieval (41, 42). Namely, before immunostaining, antigen retrieval was performed by heating thin sections in 10 mM citrate buffer (pH 6.0) for 25 min at 95°C using KOS Microwave HistoSTATION (Millestone Srl, Sorisole, Italy).

## Data Availability

The raw data from each experiment are included in the supplemental data set.

## SUPPLEMENTAL MATERIAL

Table S1, Fig. S1-S5 spectrum.00729-23-s0001.pdf Table S1, Fig. S1-S5.

Raw data spectrum.00729-23-s0002.xlsx Raw data.

ASM does not own the copyrights to Supplemental Material that may be linked to, or accessed through, an article. The authors have granted ASM a non-exclusive, world-wide license to publish the Supplemental Material files. Please contact the corresponding author directly for reuse.
