# Supplementary material for: Development of monoclonal antibodies against Rhodococcus equi virulence-associated protein N and their application to pathological diagnosis
Source: Microbiol Spectr. 2023 Oct 6;11(6):e00729-23. doi: 10.1128/spectrum.00729-23 (PMC10714782; doi:10.1128/spectrum.00729-23)
Supplement: Table S1, Fig. S1-S5 [file spectrum.00729-23-s0001.pdf]

Table S1. Summary of cases of VapN-harboring *Rhodococcus equi* infection in ruminants

Case No.	Year	Location	Case summary	Immunostained organs (abscess-forming organs)	Reference
Goat case No. 1	2015	Okinawa, Japan	A 5-year-old male goat presented with anorexia and shedding. After 2 months, the goat was projected to have a poor prognosis because of astasia. Multifocal lesions, such as necrotizing granulomatous lymphadenitis in the mesenteric lymph nodes, lymphoid follicle hyperplasia in the spleen, and granular degeneration in the liver, were found via pathological examination.	Colonic lymph nodes<br>Mesenteric lymph nodes	(10)
Goat case No. 2	1998	Michigan, USA	A 1-year-old crossbred goat displayed had diffuse skeletal muscle atrophy with increased laxity in the right foreleg and lateral subluxation of the right glenohumeral joint. Because of the extensive involvement of the bone, the goat was euthanized for necropsy. Numerous caseating granulomas were disseminated throughout the liver,	Right fifth rib	(24)

			lungs, abdominal lymph nodes, medulla of the right humerus, and right fifth rib.		
Cattle case No. 1	2017	Oita, Japan	A 19-month-old Japanese black presented with draining pus and enlarged subiliac lymph nodes. Three months later, respiratory symptoms were also noted, and the prognosis was considered poor. The animal was euthanized and necropsied. Granuloma formation with xerostomia necrosis was observed in the lungs, abomasum, and lymph nodes throughout the body.	Abomasum<br>Internal iliac lymph nodes<br>Subiliac lymph nodes<br>Lungs	This study
Cattle case No. 2	2022	Aichi, Japan	A 14-month-old Japanese black presented with hindlimb paralysis and an inability to stand. The prognosis was considered poor, and the animal was euthanized and necropsied. Granuloma formation with xerostomia necrosis was observed in the lymph nodes throughout the body.	Mesenteric lymph nodes	This study
Cattle case No. 3	2022	Gifu, Japan	A 43-month-old Japanese black presented with a mass in the abdominal cavity during	Lungs<br>Retropharyngeal lymph	This study

rectal examination. Nine months later, the animal was found to have shaving, abdominal tenderness, and soluble diarrhea, and the prognosis was considered poor. The animal was euthanized and necropsied. Granuloma formation with xerostomia necrosis was observed in the lungs and lymph nodes throughout the body.

nodes

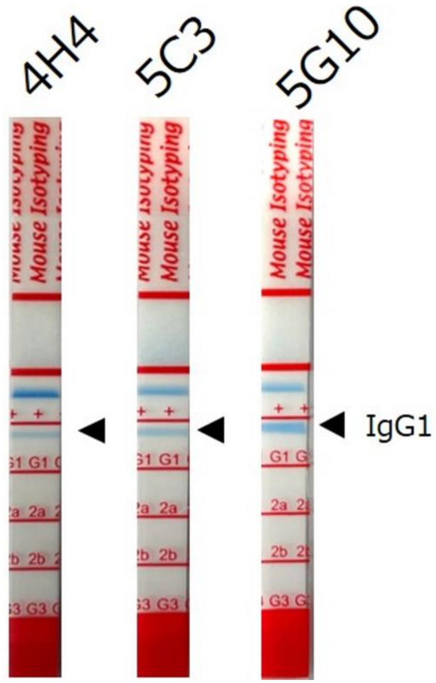

Fig. S1 Determination of the IgG subclass of each purified antibody using the Mouse Monoclonal Antibody Isotyping Test Kit (Bio-Rad).

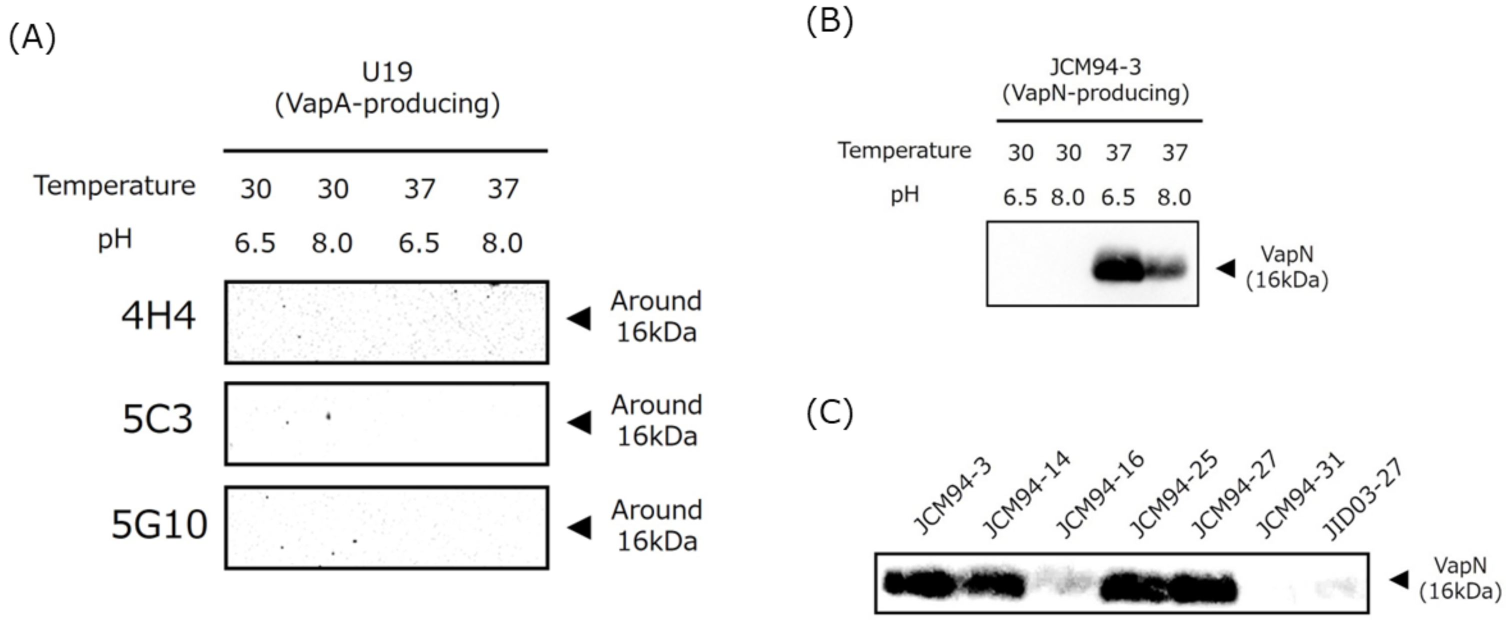

Fig. S2 Western blotting against *R. equi* lysates using the anti-VapN monoclonal antibody. (A) Western blotting was performed using a lysate of a different VapA-producing *R. equi* strain (strain U19) from the strain analyzed in Fig. 2B. In this study, we confirmed that the U19 strain does not react with the anti-VapN monoclonal antibody. (B) In comparison to the conditions in Fig. 2B, the exposure time was shortened, and the antibody concentration was reduced (1st antibody: 4H4 monoclonal antibody at 1 µg/ml, 2nd antibody: × 50,0000). The band appears notably stronger in the 37°C/pH 6.5 culture compared to the 37°C/pH 8.0 culture. The denser bands are likely a result of antibody concentration saturation observed in Fig. 2B. Interestingly, VapN production in the 30°C culture, which was previously undetectable by mouse immune serum or other monoclonal antibodies used, can be detected at the same concentration, indicating that the 4H4 monoclonal antibody is highly sensitive to VapN. (C) Three VapN-producing strains (JCM94-3, JCM94-25, and

JCM94-27), three VapN-nonproducing strains (JCM94-16, JCM94-31, and JID03-27), and one weakly VapN-producing strain (JCM94-14) were incubated at 37°C, pH 6.5, and cell lysate preparation was performed as described previously (12,15). Bands were detected in JCM94-3, JCM94-25, and JCM94-27. However, JCM94-14 exhibited a relatively thinner band, and there were no bands in the remaining three strains. These results are consistent with previous findings using VapN-immunoserum (15). These results support the existence of “strains that do not produce VapN” among “genetically VapN-positive” strains.

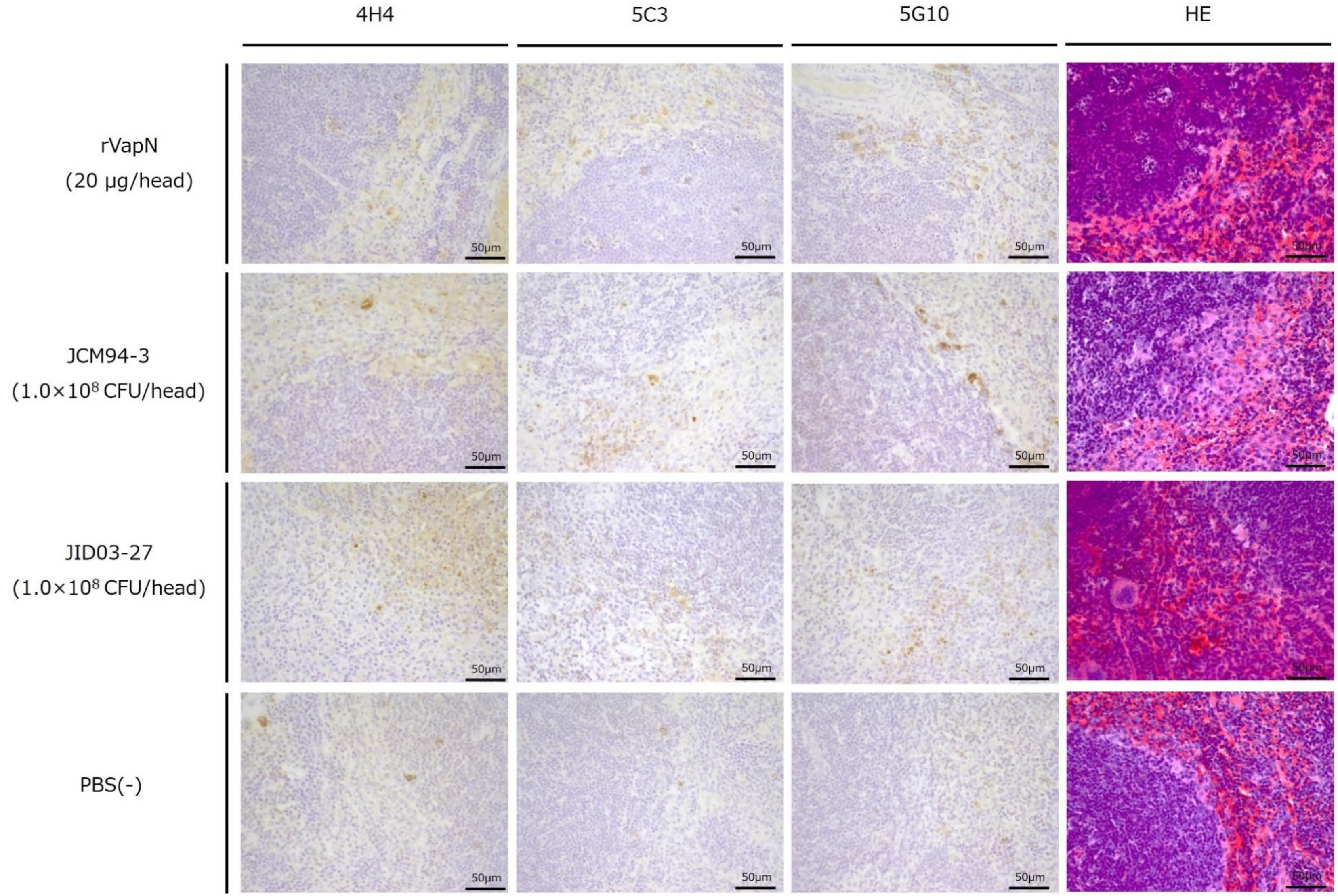
4H4
5C3
5G10
HE
rVapN
(20 μg/head)
JCM94-3
(1.0×10^8 CFU/head)
JID03-27
(1.0×10^8 CFU/head)
PBS(-)
50μm

Fig. S3 Pathological changes in the spleen after the inoculation of mice with rVapN, a VapN-producing strain (JCM94-3), or a VapN-lowproducing strain (JID03-27). Administration of rVapN or the VapN-producing strain resulted in numerous “starry sky-like patterns” of necrosis in large cells (presumably reticulocytes) in white pulp, with positive immunostaining reactions detected in the center of these cells. Conversely, positive reactions in the border region between the white pulp and red pulp of the spleen were detected in all mice including the PBS (−)-administered mice, suggesting that these positive reactions were nonspecific. Bar = 50 μm.

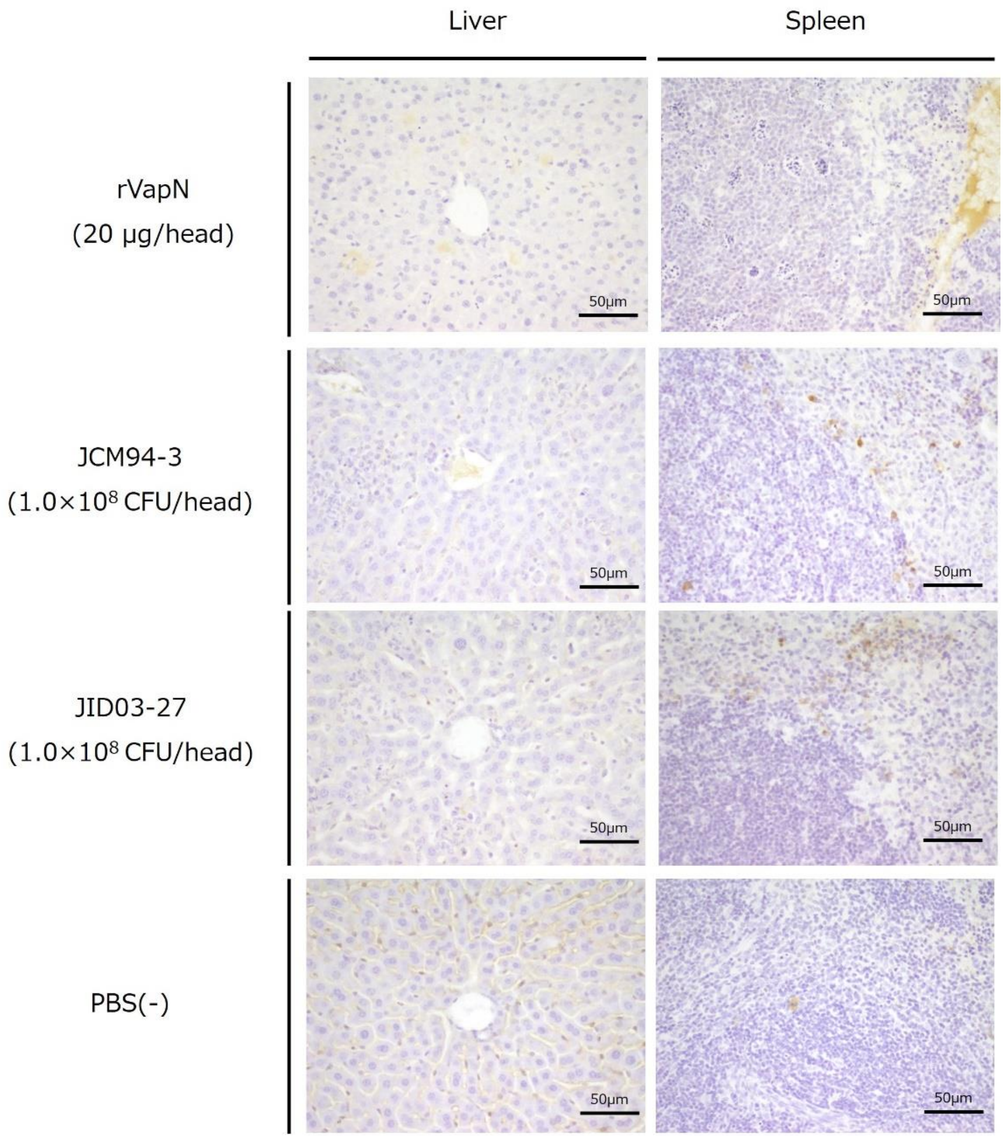
Liver
Spleen
rVapN
(20 µg/head)
JCM94-3
(1.0×10^8 CFU/head)
JID03-27
(1.0×10^8 CFU/head)
PBS(-)
50µm

Fig. S4 Negative controls in the VapN immunostaining method using mouse models. The same tissues used in Fig. 3 (liver) and Fig. S3 (spleen) were immunostained using commercially available mouse IgG as the primary antibody. In the liver, there was no positive reaction in single-cell necrosis and microgranuloma that was stained using the anti-VapN monoclonal antibodies. In the spleen, there was no positive reaction in the center of the “starry sky-like image” stained using the monoclonal antibodies. The reaction at the boundary between the white and red splenic medulla was nonspecific, similar to when monoclonal antibodies were used. Bar = 50 μm.

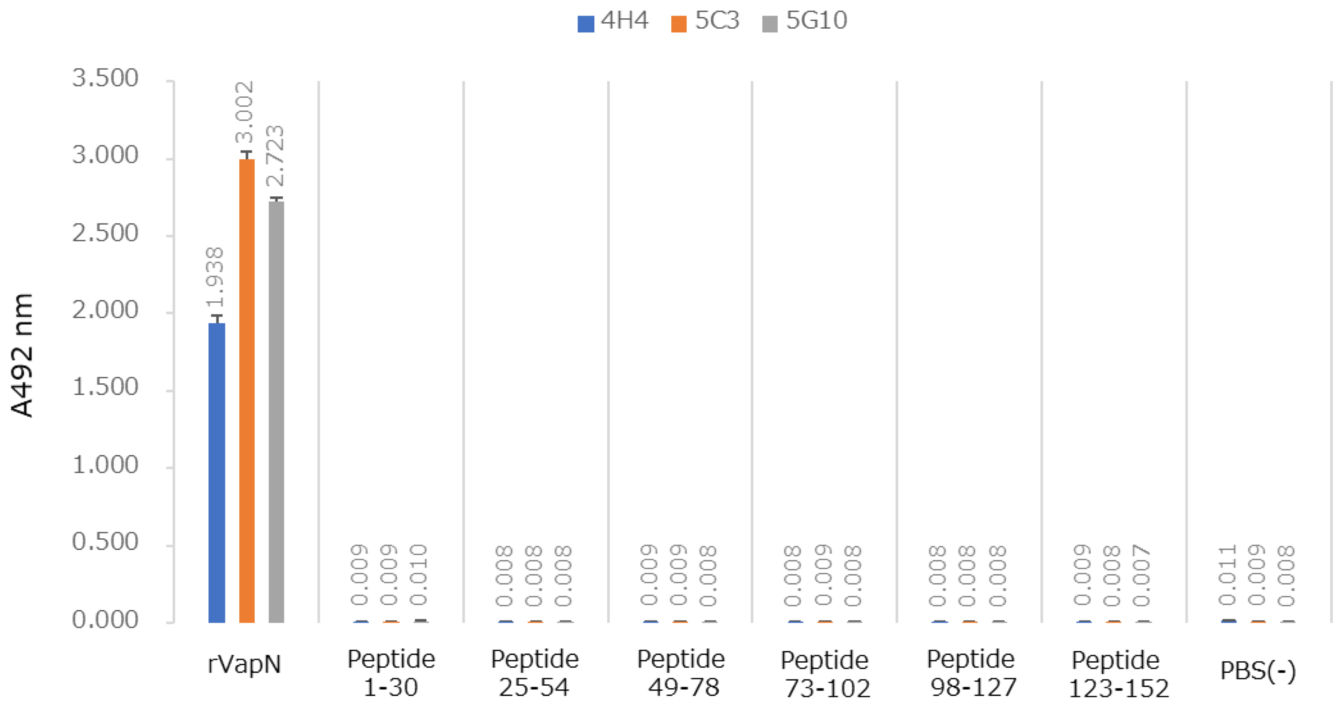

Fig. S5 Each monoclonal antibody reacted to a six-peptide fragment of VapN. At 4°C overnight, rVapN or each peptide was immobilized at 100 ng/well. The numbers above the bar are the mean of A492nm readings. The experiments were conducted in quadruple, and results are provided as mean ± SEM.
